# Plasmonic probing of the adhesion strength of single microbial cells

**DOI:** 10.1073/pnas.2010136117

**Published:** 2020-10-15

**Authors:** Yi-Nan Liu, Zhen-Ting Lv, Wen-Li Lv, Xian-Wei Liu

**Affiliations:** ^a^Chinese Academy of Sciences Key Laboratory of Urban Pollutant Conversion, Department of Environmental Science and Engineering, University of Science and Technology of China, 230026 Hefei, China;; ^b^Department of Applied Chemistry, University of Science and Technology of China, 230026 Hefei, China

**Keywords:** microbial adhesion, plasmonic imaging, interfacial force, biofilm formation

## Abstract

Probing the binding between a microbe and surface is critical for understanding biofilm formation processes, developing biosensors, and designing biomaterials, but it remains a challenge. Here, we demonstrate a method to measure the interfacial forces of bacteria attached to the surface. We tracked the intrinsic fluctuations of individual bacterial cells using an interferometric plasmonic imaging technique. Unlike the existing methods, this approach determined the potential energy profile and quantified the adhesion strength of single cells by analyzing the fluctuations. This method provides insights into biofilm formation and can also serve as a promising platform for investigating biological entity/surface interactions, such as pathogenicity, microbial cell capture and detection, and antimicrobial interface screening.

## Significance

Studying interfacial dynamics of a single biological entity (cell, virus, or organelle) is critical for understanding microbial biofilm formation processes, developing biosensors, and designing biomaterials. However, the ability to measure the binding force between a single biological entity and surface remains a great challenge due to the hydrated feature of biological entity. In this paper, we present an optical imaging method that is able to measure the adhesion strength of single microbial cells. Unlike the existing methods with limited throughput, this method determines the adhesion strength of multiple individual cells simultaneously. This approach has the potential to contribute to a better understanding of the adhesion process at the microscopic scale and probe the biointerfaces.

Microorganisms can form biofilms, which are widely distributed and present on biotic and abiotic surfaces in natural, industrial, and medical settings (1–3). Initial bacterial adhesion to surfaces is the most crucial step in biofilm formation. The transition from initial weak, reversible interactions between a bacterium and a surface to irreversible adhesion involves complex physicochemical forces, including specific receptor-ligand forces, nonspecific hydrophobic, and electrostatic forces (4). Understanding and managing bacterial adhesion, especially at single-cell level, is a cross-disciplinary challenge (5, 6).

While many methods have been developed for study of bacterial adhesion, most technologies are based on ensemble analysis of a vast population of cells, which washes out heterogeneity and microscopic information of single bacterial cells, and cannot measure the forces driving cell adhesion. Several methods are now available to study bacterial adhesion at the single-cell level (7–9). For example, atomic force microscopy (AFM) measures interfacial forces by mechanically moving one cell with the AFM probe (7, 10). Optical tweezers are another force spectroscopy method with an intense laser field that uses microbeads attached to the cell (11, 12). These methods measure one single cell at one time, thus having limited throughput. Additionally, they exert external forces on the cell and interfere with the intrinsic feature of bacterial adhesion.

Here, we aim to probe the interfacial forces by measuring intrinsic fluctuations of bacteria attached to the surface using plasmonic interferometric imaging technique. Unlike AFM or optical tweezers, this method enabled us to perform high-throughput tracking of many single bacterial cells, to determine the potential energy profile for each bacterial cell and obtain the elastic parameters. To probe the tiny vertical fluctuations, we imaged the interferometric pattern of bacterial cells scattering the planar plasmonic wave propagating on the surface. The plasmonic scattering intensity was extremely sensitive to the vertical distance, allowing precise tracking of the fluctuations. From the fluctuation analyses, we obtained the interfacial energy profiles and elasticity of microbial binding, which were essential properties in understanding microbial adhesion. The derived binding constant can be used to quantify bacterial adhesion strength. Thus, the knowledge obtained can help understand biofilm formation and be used in the design of artificial surfaces to minimize or maximize bacterial adhesion.

## Results and Discussion

Upon bacterial attachment to the surface, weak interactions occurred between the surface and cell envelope, which allowed the vibration of the bacterial cell (5, 13, 14). For interaction between a single bacterial cell and a surface, the interfacial energy between the cell and the surface determines the binding strength and binding event rate and is thus important for understanding attachment processes (15, 16). The interfacial energy [*Φ*(*h*)] is based on the probability density [*P*(*h*)] according to Maxwell–Boltzmann equation (17)

Φ(h)=-kT(ln(P(h))+C), [1]

where *h* is the vertical distance of a cell from the surface, *C* is an unknown constant, *T* is temperature, and *k* is the Boltzmann constant. The probability density function *P*(*h*) can be obtained by kernel density estimation of the bacterial vertical position.

**Fig. 1.**
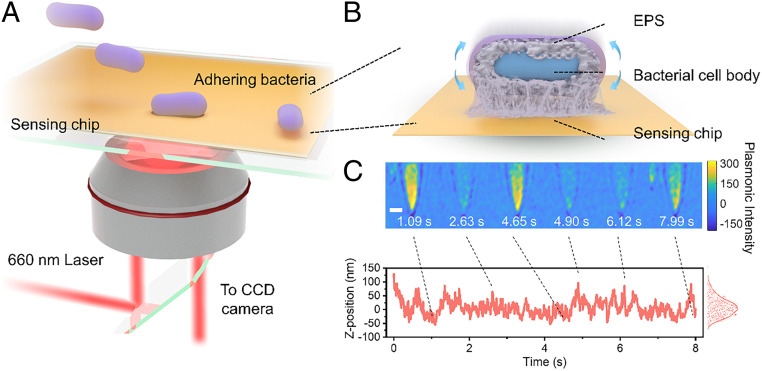
Imaging of the vertical fluctuation of adhered bacteria. (*A*) Setup of the plasmonic interferometric imaging system. (*B*) Schematic diagram of the interface between the bacterial envelope and surface. (*C*) Time-resolved plasmonic images of a single adhering bacterial cell (*Upper*) and the corresponding vertical distance fluctuation (*Lower*). The *Z*-position distribution of the bacterium is shown in the right histogram. (Scale bar in *C*, 2 μm.)

To probe this fluctuation, we used a plasmonic interferometric imaging technique based on an inverted microscope with a high-numerical-aperture oil-immersion objective (Fig. 1*A*). A glass coverslip coated gold film was placed on the objective. Incident light from a superluminescent diode was directed onto the gold film from the objective to excite surface plasmons (18–20). Scattering of the plasmonic waves by individual bacterial cells on the surface generated interferometric single-cell images with high contrast, which were recorded by a charge-coupled device camera. The plasmonic scattering intensity of the bacterial cell decreases exponentially as the distance between the cell and surface (21, 22). This feature enables a sensitive determination of the vertical displacement, *Z*, of individual bacterial cells. The relationship between the plasmonic intensity and vertical displacement is

$$I_{\text{z}}=I_{0}\exp\left(-\frac{Z}{L}\right),$$ [2]

where *I*_0_ is the plasmonic intensity of the bacterium at *Z* = 0, *L* is the decay length constant of the evanescent field (100 nm) (23).

We loaded the bacterial solution onto the sensing chips and determined the vertical fluctuations by recording the time-resolved plasmonic images using Eq. **2**. Here, *Sphingomonas wittichii* RW1, a model strain for studying bacterial adhesion, was used (24). The video of plasmonic imaging of the fluctuations of bacteria is presented in the *SI Appendix* (Movie S1). Fig. 1*C* shows several snapshots from a video of a single bacterial cell and the time profile of the vertical fluctuations from the plasmonic images over time.

**Fig. 2.**
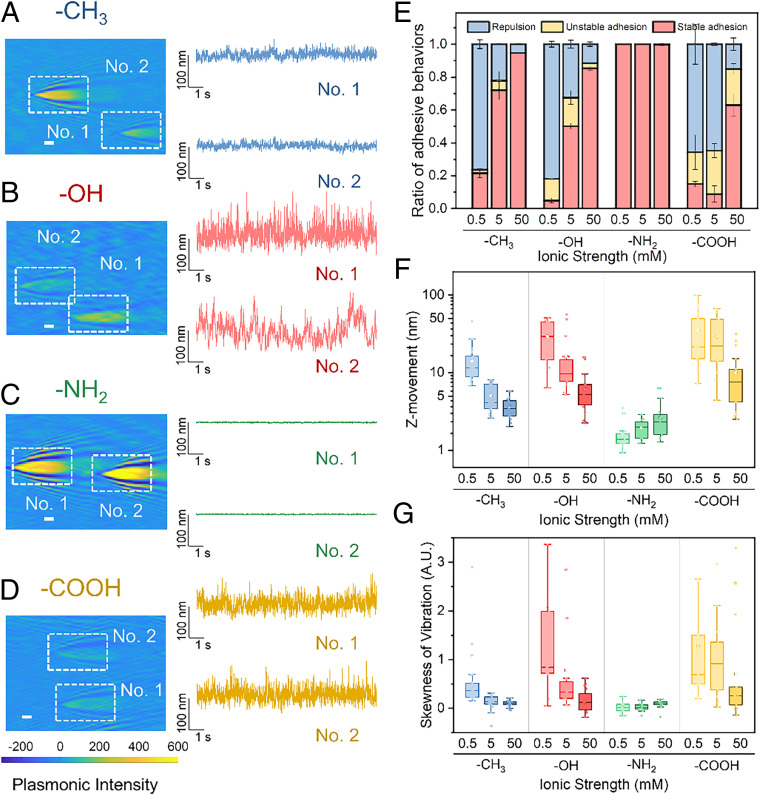
Differences in the fluctuation features of bacteria with different adhesion strengths. (*A*–*D*) Plasmonic images and vibration profiles of bacteria in 0.5 mM KCl solution on four kinds of SAM-coated surfaces: C_11_-CH_3_ (*A*), C_11_-OH (*B*), C_11_-NH_2_ (*C*), and C_11_-COOH (*D*). (*E*) Ratio of bacterial adhesion behaviors in different conditions. The amplitude (*F*) and skewness (*G*) of bacterial fluctuation in various conditions. (Scale bars in *A*–*D,* 2 μm.) For boxplots in *F* and *G*, the centerlines indicate medians, box bodies contain the data from the first to third quartile, the whiskers extend to a 1.5× interquartile range, and the dots represent the data. The error bars in *E* and figures below indicate the SD of the data.

To study the fluctuation features of bacteria under different surface and solution chemistry conditions, we functionalized the gold surface using self-assembled monolayers (SAMs) with four different ending functional groups (-NH_2_, -COOH, -CH_3_, and -OH) and varied the ionic strength of the solution (0.5, 5, and 50 mM). This strategy induced different surface potential and surface chemistry, allowing for variations in bacterial adhesion features. We found that the hydrophobic/less-negative charged surfaces (ending with -CH_3_ or -NH_2_) and a high ionic strength (e.g., 50 mM) facilitated irreversible (stable) bacterial adhesion. In contrast, the hydrophilic/more-negative charged surfaces and low ionic strength (ending with -OH or -COOH and the ionic strength of 0.5 mM) resisted this irreversible bacterial adhesion, and most of the cells were repelled (Fig. 2 *A*–*E*, Movie S2, and *SI Appendix*, Figs. S1 and S2).

We quantified the fluctuation amplitudes (for stable and unstable adheres) in different adhesion conditions and found that they are associated with the binding energy (Fig. 2*F*). The vibrational amplitudes of bacteria in adhesion-resistant conditions were much larger than those in adhesion-favorable conditions. For the bacteria on the -OH and -COOH surfaces at an ionic strength of 0.5 mM, the adhesions were severely weakened. Their fluctuation amplitudes were 30.8 ± 15.0 nm and 10.6 ± 9.1 nm, respectively. For bacteria on the -CH_3_ or -NH_2_ surfaces at an ionic strength of 50 mM, the amplitudes were 3.6 ± 1.1 nm and 2.9 ± 1.6 nm, respectively (Fig. 2*F*). We then quantified the skewness of bacterial vertical position distributions (Fig. 2*G*), which exhibited features similar to a fluctuating amplitude. With the weakening of adhesion, the skewness of vibration sharply increased (from ∼0–0.92).

**Fig. 3.**
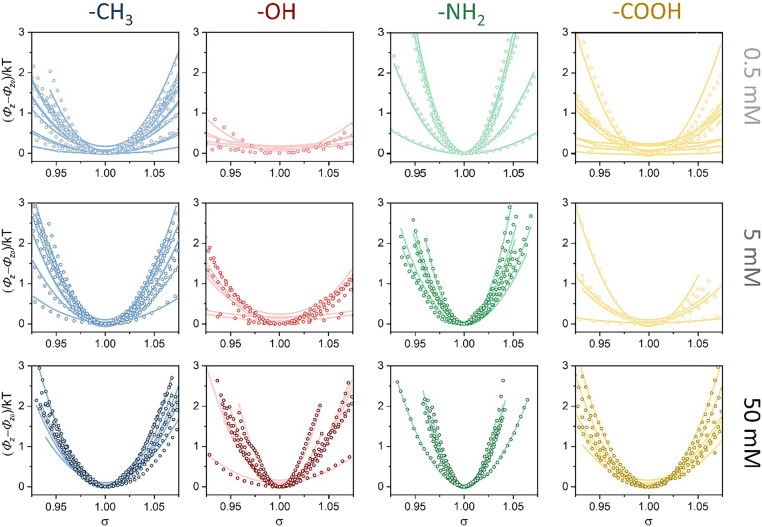
Bacterium-surface potential energy profiles. The potential energies derived from bacterial cell *Z*-position fluctuations in different adhesive conditions are shown in circle and the polymer elasticity fittings are demonstrated by lines.

The very large difference in the fluctuation implied that there are distinct interfacial energy profiles. We next determined the single-cell adhesion potential profiles using Eq. **1** (Fig. 3). The steep potential curves corresponded to the fluctuation activity. The gentle potential curve indicates that the bacteria were active during the fluctuation. For an ideal harmonic vibration, the potential distribution could be symmetric near equilibrium (16). However, we observed asymmetry when we zoomed in on the time profiles of the vertical fluctuations and potential-distance relationships (Figs. 2 and 3). Such biased potential energy distribution implies a nonlinear deformation–force relationship, which is completely different from abiotic oscillators tethered with DNA or other linkers (17).

The outermost surface of bacteria was covered by a layer of extracellular polymeric substances (EPS) which was a polymeric network, composed of polysaccharides, proteins, and DNA. Due to their polymer nature, EPS can create bridges between bacteria and surface, and tether bacteria from leaving (25). The EPS layer exhibited alternate tension and compression due to the Brownian motion of adhering bacteria, and its viscoelasticity introduced a nonlinear component into the binding potential. Given the viscoelastic nature of EPS, the binding stress of adhering bacteria can be described by the stress–strain relationship of the polymer (26)

$$\sigma =G\left(\alpha -\frac{1}{\alpha^{2}}\right),$$ [3]

G=νRT, [4]

$$\alpha =\frac{h}{l_{0}},$$ [5]

where *G* refers to the elastic modulus of the polymer network; ν is the concentration of the elastically effective chain that is proportional to the cross-linking degree of the polymer; *R* is the ideal gas constant; and *l*_0_ is the initial length of the elastomer. The elastic potential energy of the elastomer [Δ*Φ*(*h*)] can be obtained by integrating Eq. **3**:

$$\Delta \Phi \left(h\right)=\int_{l_{0}}^{h}S_{\text{cell}}\sigma dh=K\left(\frac{h^{2}}{2l_{0}^{2}}+\frac{l_{0}}{h}-\frac{3}{2}\right),$$ [6]

$$K=GS_{\text{cell}}l_{\text{0}},$$ [7]

where *S*_cell_ is the contact area of bacterium–surface binding, and the binding constant (*K*) is defined as the product of the elastic modulus, the initial elastomer length, and contact area.

**Fig. 4.**
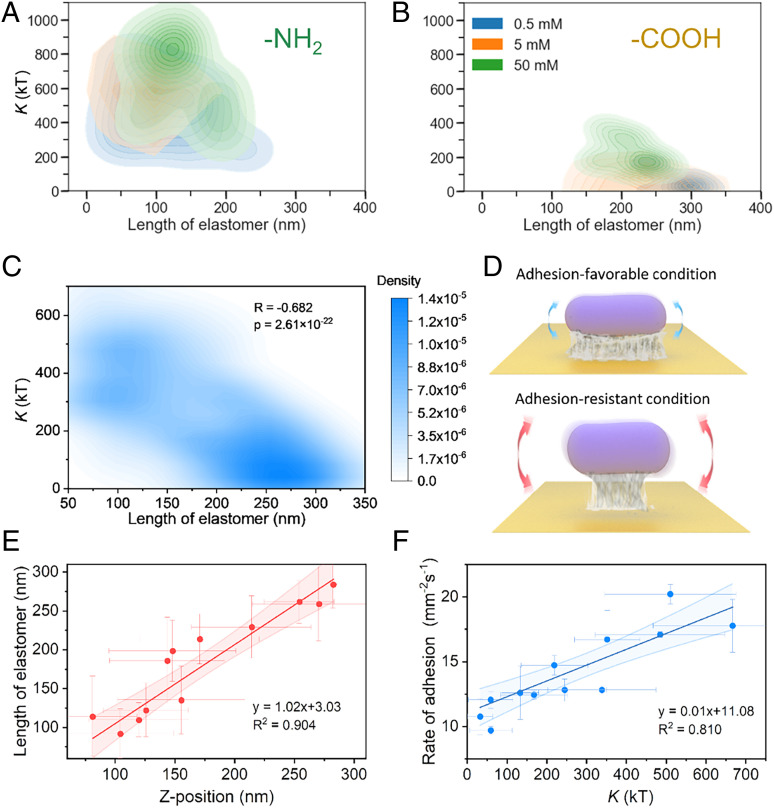
Binding elastic parameters for the quantification of the bacterial adhesion. (*A*–*D*) Binding elastic parameters of bacteria in different solutions (0.5, 5, and 50 mM KCl) on (*A*) C_11_-NH_2_- and (*B*) C_11_-COOH-coated surfaces. (*C*) Correlation of the binding constant and elastomer. (*D*) Schematic diagram of tightly and loosely binding bacteria. (*E*) Correlation of the *Z* position of bacteria and the fitting results of the elastomer length. (*F*) Adhesion rate under different adhesion conditions as a function of the binding constant. The darker-shaded regions in *G* and *F* indicate the 95% confidence interval of the fitting.

Using Eqs. **6** and **7**, we quantified the energy profiles (Fig. 3) and extracted two elastic parameters, the binding constant and elastomer length, which could be used to quantitatively determine the single-cell binding strength. The binding constant was related to the elastic modulus and the contact area, and the length of the elastomer was the initial length of the polymer network without an external force. For the bacteria on the -NH_2_ surface, the binding constants and elastomer lengths were insensitive to the ionic strength (Fig. 4*A*). The cells have a high binding constant (∼500 *kT*) and low elastomer length region (∼100 nm), indicating a high binding strength. In contrast, for cells on the -COOH, -CH_3_, and -OH surfaces, the binding constant and elastomer length exhibited a dependence on ionic strength. The increased ionic strength improved the binding force, with increased binding constants and decreased elastomer lengths of bacterial cells (Fig. 4*B*, and *SI Appendix*, Figs. S4 and S5). Although the cells were heterogeneous, the distributions of bacterial cells were located at low binding constants and high elastomer lengths compared with those on the -NH_2_ surface. Since the -COOH, -CH_3_, and -OH surfaces and bacterial cell surface were negatively charged (27), the electrostatic repulsive force prevented the formation of strong bacterium–surface bindings probed by the plasmonic measurement of the energy profiles of single bacterial cells. The electrostatic attractive force between the positively charged -NH_2_ surface and bacteria enhanced the attachment between them. The binding constant was related to the elastomer length (*P* = 2.61 × 10^−22^, one-way ANOVA; Fig. 4*C*), which could be attributed to the decrease in the binding strength as the elastomer length increased.

To verify the extracted parameters for the quantification of the binding strengths, we compared the adhesion results with the binding constants and elastomer lengths. The elastomer lengths were consistent with the equilibrium separation distances of the bacteria (*R*^2^ = 0.904; slope = 1.01 ± 0.10; Fig. 4*E*), indicating that EPS were responsible for the binding to the surface. More importantly, the binding constants were positively related to the bacterial adhesion rates (Fig. 4*F* and *SI Appendix*, Fig. S6) and negatively related to the dissociation constants of the bacterial detachment from the surface (*SI Appendix*, Fig. S7). We therefore correlated the binding strength of a single cell at a microscopic scale with the adhesion rate at a macroscopic scale, filling the knowledge gap regarding the contribution of both single bacterial cells and communities of bacterial cells to adhesion. Therefore, the binding constant could be used to effectively quantify the adhesion strength.

**Fig. 5.**
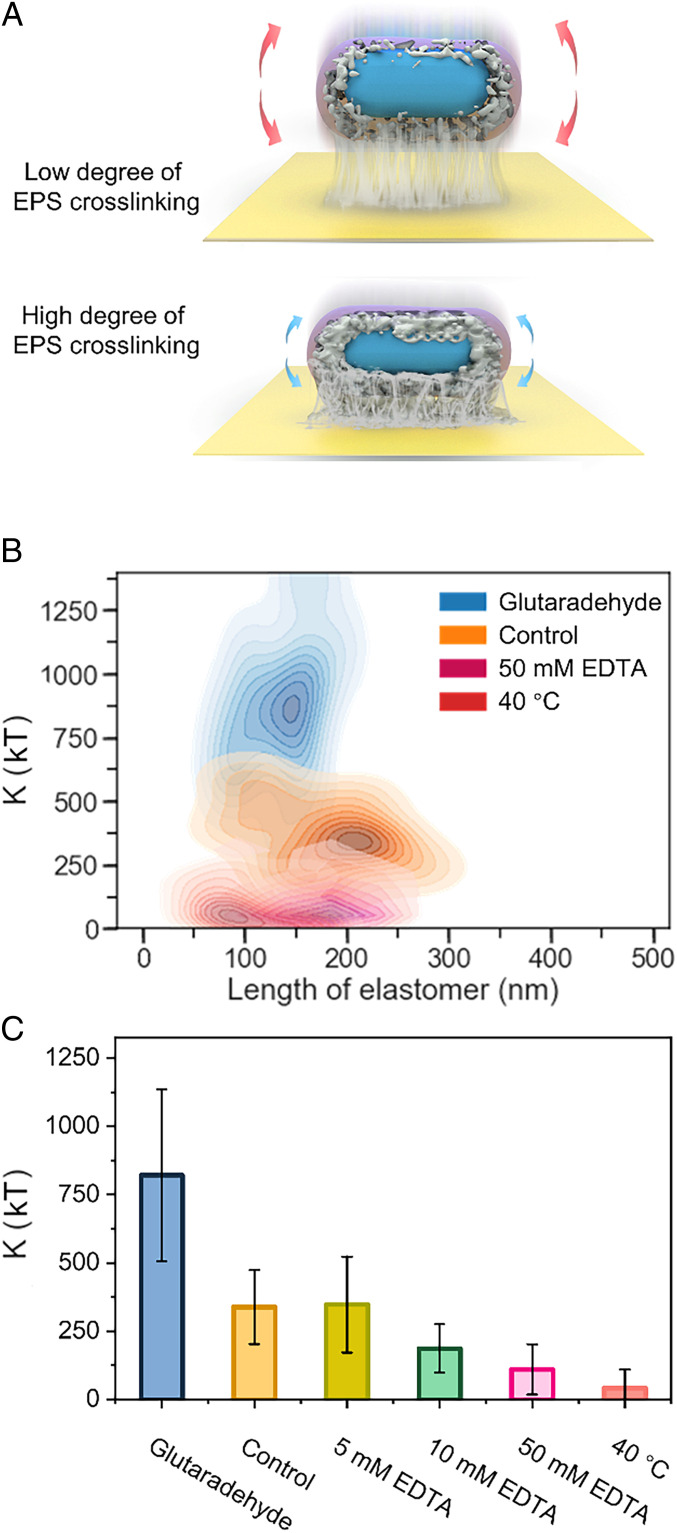
Verification of the method for quantifying bacterial binding strength. (*A*) Schematic diagram of bacteria with different degrees of EPS cross-linking. (*B*) Binding elastic parameter of bacteria after EPS-related treatments. (*C*) Binding constant of bacteria after EPS-related treatments.

Bacterial binding was highly dependent on EPS. We therefore hypothesized that the binding constant is proportional to the elastic modulus of the EPS network and that the change in network structure can influence the vibration profile (Fig. 5*A*). To verify this hypothesis, we conducted experiments to modulate the cross-linking degree of EPS. We used two methods to reduce the network cross-linking, which included ethylenediaminetetraacetic acid (EDTA) and a heating treatment, in order to chelate the divalent cations from EPS or disassemble EPS (28). We also used the crosslinking agent glutaraldehyde to increase the rigidity of the EPS network. After the EPS treatment, the binding constants changed considerably (Fig. 5 *B* and *C*). The changes in degree of cross-linking altered the elastic modulus of EPS, which led to variations in the binding constant. We also digested the extracellular polysaccharides of *S. wittichii* RW1 with amylase, which could directly influence the bacterial binding strength. After the amylase treatment, the binding constant of bacteria was enhanced from 324.4 ± 44.4 *kT* to 515.2 ± 72.2 *kT* (*SI Appendix*, Fig. S9). The distribution of bacterial binding constant after the amylase treatment was wider, indicating that the bacterial cells were heterogeneous and some of the bacterial cells were more resistant to changes in the mechanics.

## Conclusions

In summary, we have demonstrated a method to measure the microbial adhesion strength by plasmonic probing of the vertical fluctuations of single cells in a heterogeneous population. By analyzing the amplitude and distribution of fluctuations, we determined the binding constant and elastomer length, which could be effectively used to quantify the adhesion strength of single cells. This work is a proof-of-concept demonstration of a high-throughput measurement of the microbial adhesion strength without external interference. We anticipate that this method will contribute to understanding of the adhesion process at the microscopic scale and could be applied to the attachment and infection of single viral/bacterial cell to host cells, single-molecule targeting membrane proteins, and high-throughput screening techniques, and the sorting of antifouling interfaces.

## Materials and Methods

### Experimental Setups and Bacterial Culture.

The plasmonic imaging setup was built on a total internal reflection microscope (Ti-E, Nikon, Japan). Gold-coated coverslip was used as the sensing chip. Before use, the chips were functionalized using the SAMs with four different ending functional groups (-NH_2_, -COOH, -CH_3_, and -OH). *S. wittichii* RW1 (CICC10426) was incubated at 30 °C until early stationary phase and washed twice with KCl solution of different concentrations (0.5, 5, or 50 mM) before adhesion experiments. More details are provided in *SI Appendix*.

### Bacterial Adhesion Probing.

To determine the adhesion behavior and the vertical fluctuation of single bacterial cells, we recorded the plasmonic images at the frame rate of 7 and 106 frames per second during each adhesion experiment, respectively. The plasmonic intensity of single cell was extracted from the recorded image sequences. The intensity was further converted into the vertical position of bacteria for energy analysis. The data processing was performed using Origin 2018b (OriginLab Corporation) or MATLAB (2018b, The MathWorks) software.

## Supplementary Material

Supplementary File

Supplementary File

Supplementary File

## Data Availability

All data needed to evaluate the conclusions of the paper are present in the main text, Movies S1 and S2. All study data are included in the article and *SI Appendix*.
