# Supplementary material for: Plasmonic probing of the adhesion strength of single microbial cells
Source: Proc Natl Acad Sci U S A. 2020 Oct 15;117(44):27148–53. doi: 10.1073/pnas.2010136117 (PMC7959563; doi:10.1073/pnas.2010136117)
Supplement: Supplementary File [file pnas.2010136117.sapp.pdf]

Supplementary Materials for

Plasmonic Probing of the Adhesion Strength of Single Microbial Cells

Yi-Nan Liu[a], Zhen-Ting Lv[a], Wen-Li Lv[a], Xian-Wei Liu[a,b,1]

[a]Chinese Academy of Sciences Key Laboratory of Urban Pollutant Conversion, Department of Environmental Science and Engineering, University of Science and Technology of China, Hefei, 230026, China;

[b]Department of Applied Chemistry, University of Science and Technology of China, Hefei, 230026, China

[1]Corresponding authors: xianweiliu@ustc.edu.cn

www.pnas.org/cgi/doi/10.1073/pnas.2010136117

Materials and Methods

Supplementary Notes

Thermodynamic considerations on the binding constant under different adhesion conditions.

Correlation between bacterial binding constant and dissociation constant.

Supplementary Figures

Figures S1-S10

Supplementary Tables

Table S1. Parameters used for DLVO calculation.

Table S2. Calculated DLVO energy of bacteria-surface interactions.

Supplementary Videos

Video S1. Plasmonic imaging of the vertical fluctuation of single bacterial cell. Video acquired by a CCD camera at frame rate of 106 frames per second.

Video S2. Typical bacterial fluctuation behaviors at the single-cell level. Video acquired by a CCD camera at frame rate of 106 frames per second.

Materials and Methods

Bacterial strain and its growth condition. *Sphingomonas wittichii* RW1 (CICC10426) was purchased from China Center of Industrial Culture Collection (Beijing, China). *S. wittichii* RW1 was grown on nutrient agar plate containing agar (1.5 g/L), peptone (5 g/L), beef extract (3 g/L) and NaCl (5 g/L).(1) Single colony on the nutrient agar plate was transferred to the nutrient broth media containing peptone (5 g/L), beef extract (3 g/L), NaCl (5 g/L) and the culture was incubated at 30 °C until early stationary phase. Then the culture was further diluted in the nutrient broth media adding 200 μg/mL streptomycin (dilution ratio of cell culture was 1:100) and incubated at 30 °C for about 24 h to its early stationary phase. The bacteria were then harvested by centrifugation (6000 g, 5 min), washed twice in KCl solution of different concentrations (0.5 mM, 5 mM or 50 mM), respectively. We re-suspended bacterial pellets in the KCl solution for the adhesion experiment at the corresponding ionic strength. These ionic strengths were selected to minimize the potential influence of ionic strength on the bacteria activity.

Setup of the plasmonic imaging system. The plasmonic interferometric imaging system was based on a total internal reflection microscope (Ti-E, Nikon, Japan). A 660 nm, 15 mW superluminescent light-emitting diode (SLED) laser (SUPERLUM, Ireland) was used as the light source. BK7 glass coverslips coated with a 2 nm Cr adhesion layer

and 48 nm of Au were used as sensing chips. A 60×oil-immersion objective lens (NA = 1.49) with×0.46 zoom-out lens was used to observe the adhesion of single bacterial cell.

Modification of sensing chips. For the self-assembled monolayer (SAM) modification, the ethanol-washed chips were submerged in 1 mM $HS\text{-}(CH_2)_{11}\text{-}CH_3$, $HS\text{-}(CH_2)_{11}\text{-}OH$, $HS\text{-}(CH_2)_{11}\text{-}NH_2$, $HS\text{-}(CH_2)_{11}\text{-}COOH$ or poly-L-lysine hydrobromide (PLL) ethanol solution overnight to coat the chips with $\text{-}CH_3$, -OH, $\text{-}NH_2$, -COOH ending groups and lysine respectively. The SAM-modified chips were washed with deionized water and ethanol, and dried with nitrogen before use.

Bacterial adhesion experiments. The sensing chip was mounted on the stage of the microscope and a silicone cell was attched to the chip to hold buffer solution. The bacteria solution (OD_{600} = 0.1) was added into the cell. The plasmonic images were recorded by a CCD camera (Pike-032B, Allied Vision Technologies, USA) simultaneously. The standard deviation of bacterial vertical position fluctuation (Z-position) was defined as the vibrational amplitude (Z-movement). To determine the adhesion behavior of single bacterial cell and the adhesion kinetics, we recorded the plasmonic images for 10 minutes at the frame rate of 7 frames per second, and 150

adhered bacterial cells were randomly selected and analyzed for each static adhesion experiment to analyze the adhesion behavior. To track the fluctuations of single bacterial cell, we recorded images at the frame rate of 106 frames per second, and analyzed the fluctuations of 30 randomly selected adhered bacterial cells during each static adhesion experiment. The data analysis were conducted using Origin 2018b (OriginLab Corporation, USA) or custom-written Matlab (2018b, The MathWorks, USA) codes.

Superficial characterization of bacteria and sensing chips. To calculate the surface energy of *S. wittichii* RW1 and sensing chips, their contact angles were measured with water, α-bromonaphthalene and formamide using a contact angle meter (JC2000C, Shanghai Powereach Digital Technology Equipment Co., China). The contact angles of bacteria were measured using bacterial layers indirectly. Before contact angle measurement, bacterial solution were filtered through a 0.22-μm acetate cellulose membrane to form a homogeneous layer. (2) The Zeta potentials of bacteria in the KCl solution with different ionic strengths were determined using a Zetasizer ZEN3600 (Malvern Instruments Co, UK). The Zeta potential of sensing chips were measured using a SurPASS3 streaming potential analyzer (Anton Paar, Austria). All the above measurements were repeated at least six times.

Calculation of bacterium-surface interactions. Considering the amplitudes and nonlinearities of potential curves, we can rule out the contribution of electrostatic and gravity potential energy in the fluctuation potential analysis (Fig. S3).

According to the X-DLVO theory, the interactions between colloids (bacteria) and infinite surface (sensing chips) can be decomposed into components of Lifshitz-van der Waals interaction (Φ_{vdW}), Lewis acid-base interaction (Φ_{AB}) and electrostatic interaction (Φ_{EL}).

$$\Phi_{\mathrm{TOT}}(h) = \Phi_{vdW}(h) + \Phi_{AB}(h) + \Phi_{EL}(h) \qquad (2)$$

Lifshitz-van der Waals interaction can be calculated from the results of superficial characterization according to

$$\Phi_{vdW}(h) = -\frac{Ar}{6h}(1+\frac{14h}{\lambda_0})^{-1} \qquad (3)$$

where A is Hamaker constant, r is the radius of bacteria. λ_0 is characteristic wavelength (100 nm). h is the separation distance between bacteria and sensing chips. Hamaker constant is derived from Lifshitz-van der Waals free energy (ΔG_{LW}) and minimum equilibrium cut-off distance (d_0), given by

$$A = -12\pi d_0^{\,2}\Delta G_{\mathrm{LW}} \qquad (4)$$

Similarly, the Lewis acid-base interaction (Φ_{AB}) and electrostatic interaction (Φ_{EL}) are derived from the Lewis acid-base free energy (ΔG_{AB}) and electrokinetic property of bacteria and sensing chips according to (3, 4)

$$\Phi_{AB}(h) = 2\pi r \Delta G_{AB} \lambda \mathrm{e}^{\frac{d_0 - h}{\lambda}} \quad (5)$$

$$\Phi_{EL}(h) = 64\pi \left(\frac{kT}{e}\right)^2 r\varepsilon \tanh\left(\frac{e\phi_1}{4kT}\right) \tanh\left(\frac{e\phi_2}{4kT}\right) e^{-\kappa h} \quad (6)$$

in which λ is the correlation length in water (0.6 nm); the φ_1 and φ_2 are the zeta potential of bacteria and sensing chips; $1/\kappa$ represents the Debye Length. k, T and e refer to Boltzmann constant, absolute temperature and electron charge, respectively. Considering the SAMs on the chips, the actual minimum separation distance is 2.1 nm (the length of C_{12} carbon chain). (5) All other parameters used in the X-DLVO potential calculation were listed in Table S1, and the obtained X-DLVO potentials at actual minimum separation distance (Φ_{h0}) and primary energy minimum (Φ_{min1}) were listed in Table S2.

Modulation of the EPS structure. Several methods were used to modulate the structure of EPS. To decrease the crosslinking degree of EPS, EDTA with different concentrations (5 mM, 10 mM and 50 mM) was added into the bacteria solution and incubated for 30 min (6). We also used heating treatment with bacteria solution heated in 40 °C water bath for 30 min. To increase the crosslinking degree of EPS, glutaraldehyde was added into bacteria solution and incubated for 5 min. In the enzyme treatment of EPS, amylase (1 U/mL) in 1×phosphate buffered saline (pH = 6.9) was incubated with the bacteria for 1 h. After the above treatments, the bacterial pellets were

washed twice and resuspended in 50 mM KCl solution for subsequent adhesion experiments.

Desorption of adhering bacteria under lateral flow. The desorption of bacteria adhered to the surface under lateral flow was performed using a surface plasmon resonance (SPR) instrument equipped with a well-designed flow cell (4500, Biosensing Instrument Inc., USA). After the adhesion of bacteria, the flow rate of buffer solution was adjusted to 100 μl/ml, which corresponded to a shear stress of 1.14 Pa to release the adhering bacteria. The SPR intensity attenuations derived from the bacteria desorption was then fitted to the 1:1 Langmuir model according to

$$I/I_0=\exp(-k_d t) \qquad (7)$$

where I_0 is the initial SPR intensity before the bacteria desorption, I is the SPR intensity during bacteria desorption at time t, and k_d is the obtained apparent dissociation constant.

Supplementary Notes

Thermodynamic considerations on the binding constant in different adhesion conditions.

To give an insight of the single-cell binding constant from a thermodynamic view of point, we calculated the adhesion Gibbs free energy changes of bacteria based on X-DLVO theory. There was a notable difference among the X-DLVO potential profiles in different adhesion conditions (Fig. S10a-b for details). The actual minimum separation distance (Φ_{h0}) is associated with interfacial energy barrier in X-DLVO potential. We therefore correlated the actual minimum separation distance with the binding constant (Fig. S10c). The binding constant decayed exponentially with the increase in minimum separation distance. When the interfacial Gibbs free energy enhanced, the corresponding binding probability of EPS would drop exponentially, which resulted in the similar decrease of *K*.

Correlation between bacterial binding constant and dissociation constant.

To validate the reliability of the binding constant for quantifying bacteria adhesion strength, we also tested the bacteria desorption behavior under lateral flow (shear stress: 1.41 Pa). The desorption kinetics under different conditions exhibits noticeable different features (Fig. S7a and b). We fitted the SPR intensity attenuations derived

from the bacteria desorption and obtained the apparent dissociation constants. The dissociation constants of –COOH surfaces were much larger than those of $–NH_2$ surfaces and declined sharply as the ionic strength increased (Fig. S7c), indicating that the binding between bacteria and surface was weak. The correlation between the dissociation constant and binding constant (Fig. S7d; R = -0.872) confirmed that our method is reliable for adhesion strength quantification.

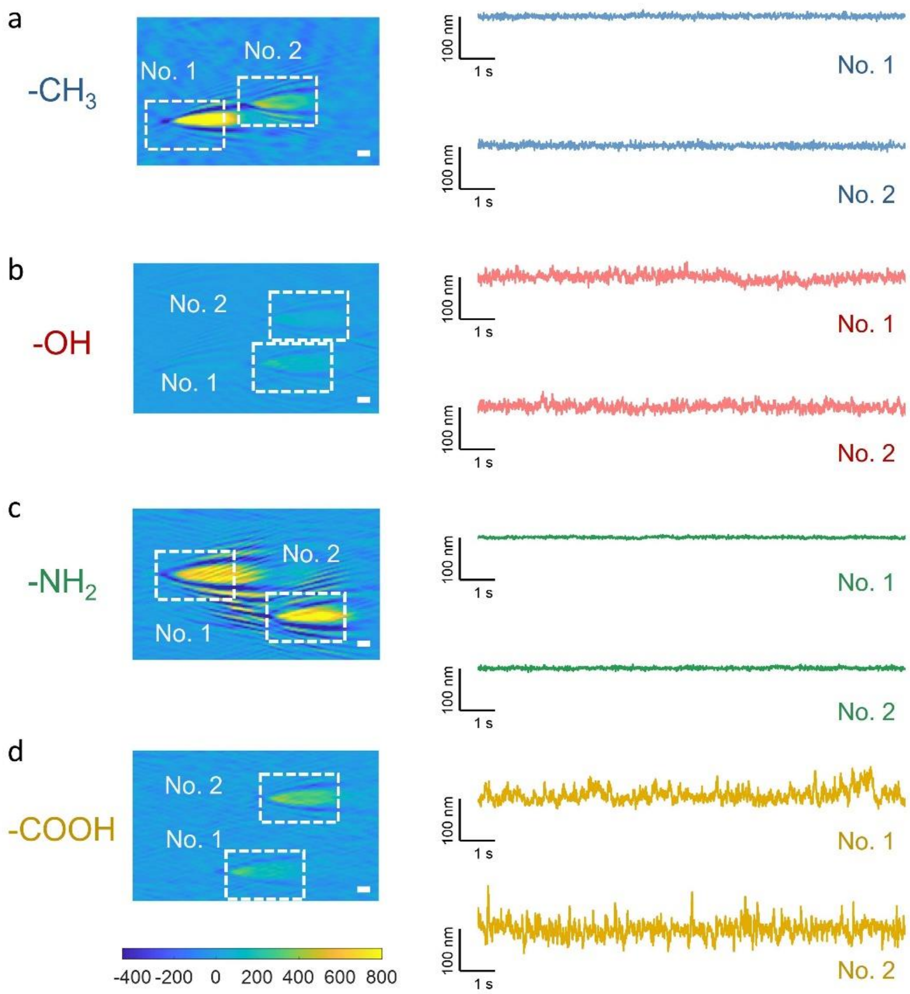

Fig. S1. Plasmonic images and vibration profiles of bacteria in 5 mM KCl solution on four kinds of SAM-coated surfaces: C_{11}-CH_3 (a), C_{11}-OH (b), C_{11}-NH_2 (c) and C_{11}-COOH (d). The scale bars in a-d are 2 μm.

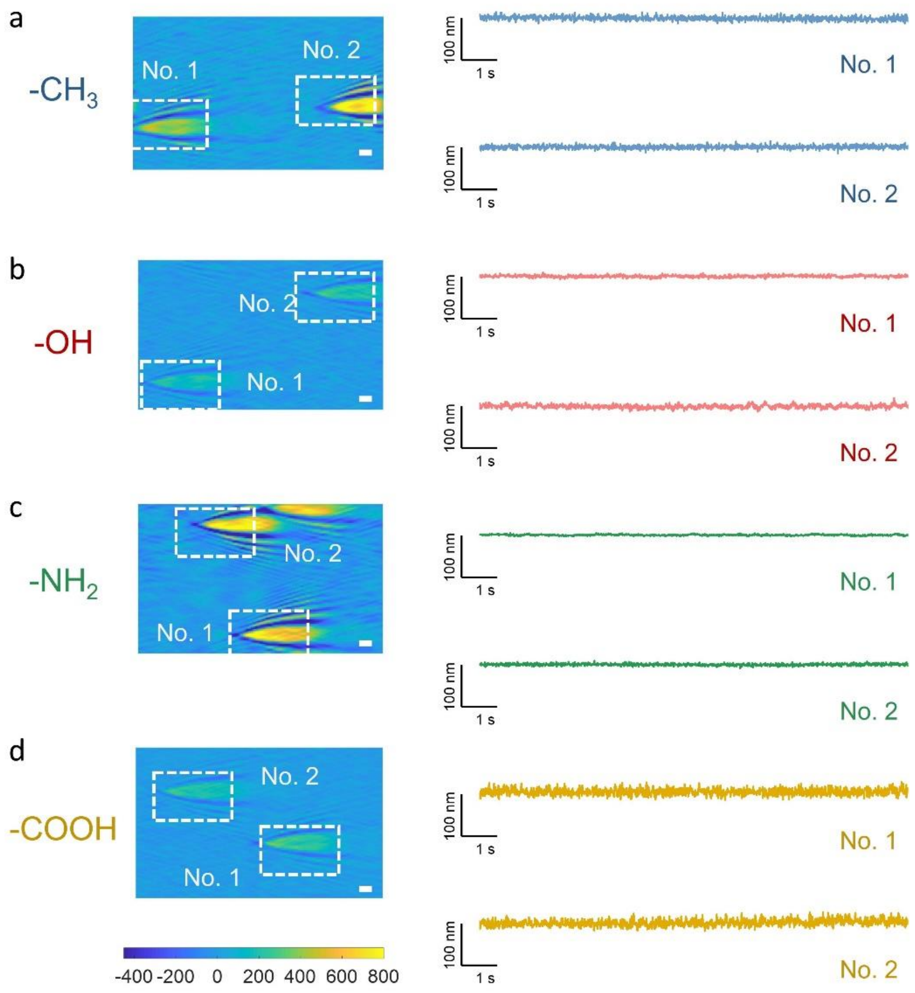

Fig. S2. Plasmonic images and vibration profiles of bacteria in 50 mM KCl solution on four kinds of SAM-coated surfaces: C_{11}-CH_3 (a), C_{11}-OH (b), C_{11}-NH_2 (c) and C_{11}-COOH (d). The scale bars in a-d are 2 μm.

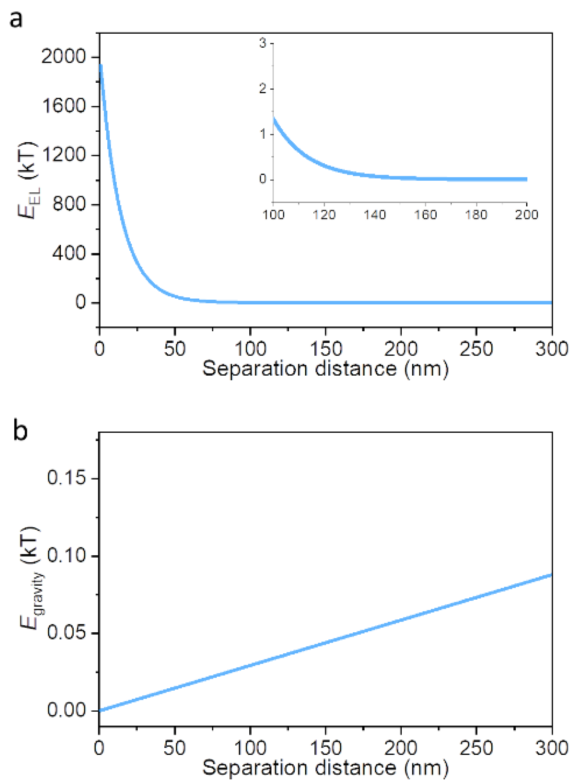

Fig. S3. Potential energy of electrostatic interaction (a) and gravity (b) of bacteria during the bacteria-surface interaction.

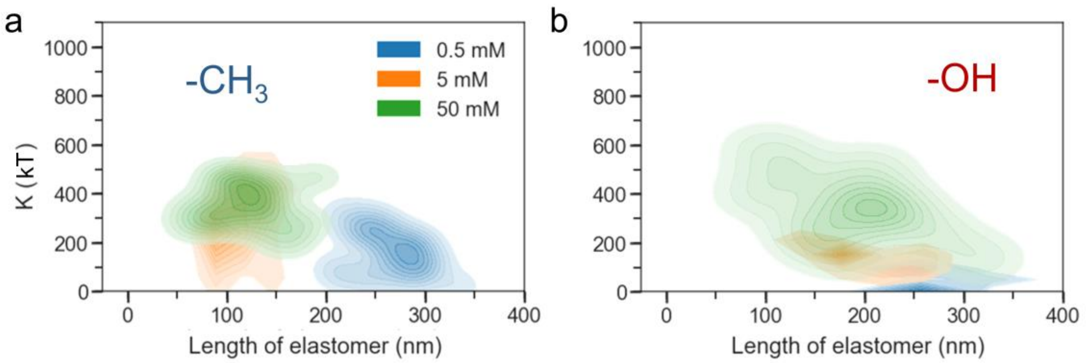

Fig. S4. Binding elastic parameters of bacteria in different solutions (0.5 mM, 5 mM, and 50 mM KCl) on C_{11}-NH_2- (a) and C_{11}-COOH-coated (b) surfaces.

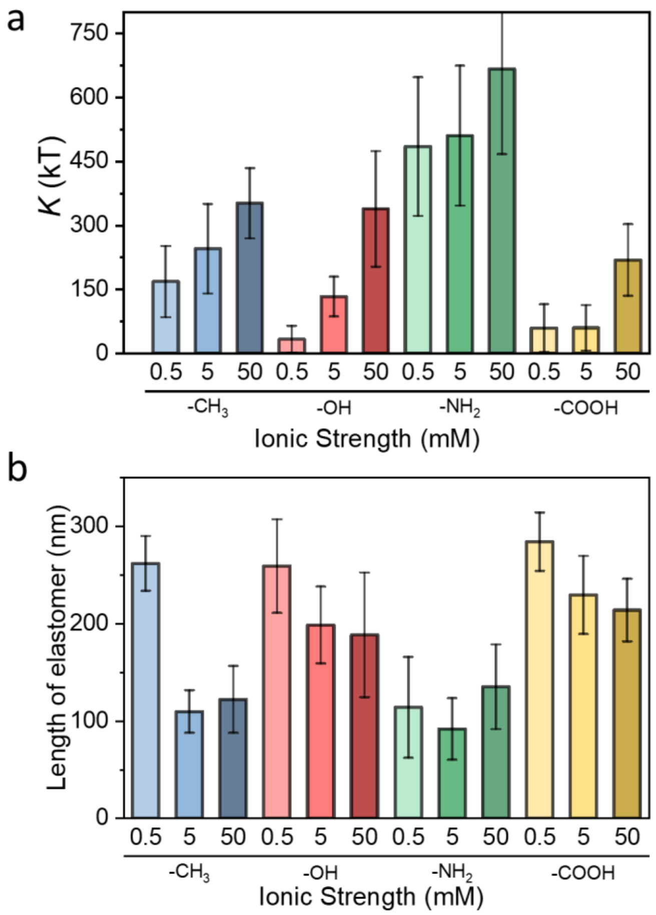

Fig. S5. Binding elastic parameters of bacteria under different adhesion conditions: binding constants (a) and elastomer lengths (b).

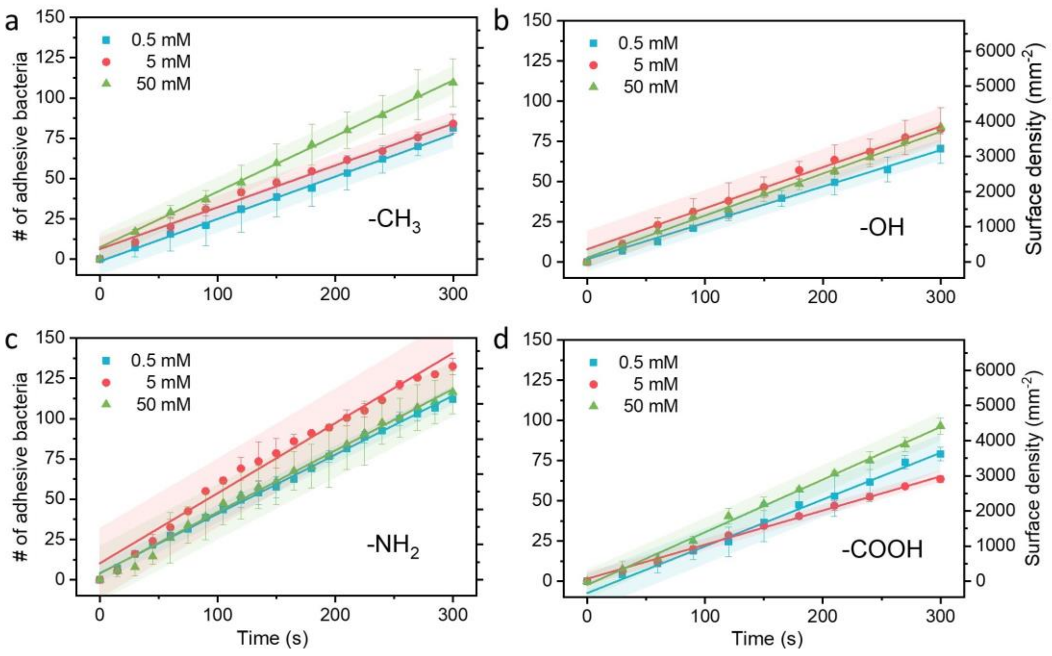

Fig. S6. Kinetics of bacterial adhesion at different ionic strengthes (0.5 mM, 5 mM, and 50 mM) on four kinds of SAM-coated surfaces: C_{11}-CH_3 (a), C_{11}-OH (b), C_{11}-NH_2 (c) and C_{11}-COOH (d). The darker shaded regions indicate the 95% prediction interval of fitting.

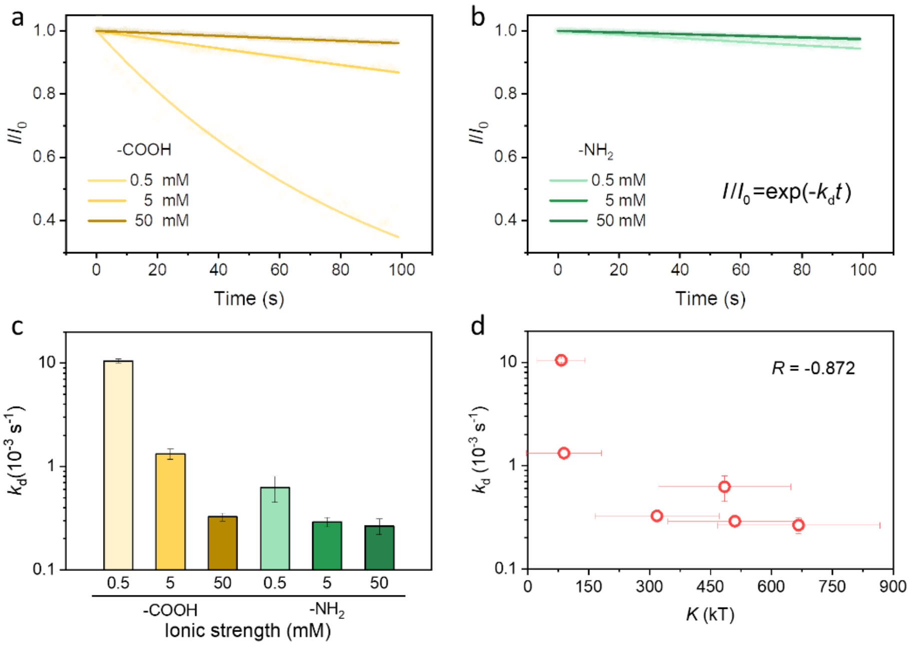

Fig. S7. Desorption of bacteria adhered to the surface under lateral flow. The desorption of bacteria under the shear stress of 1.41 Pa from -COOH surface (a) and from $-NH_2$ surface (b). The dissociation constants of bacteria under different adhesion conditions (c). The correlation between the dissociation constant (k_d) and binding constant (K) (d).

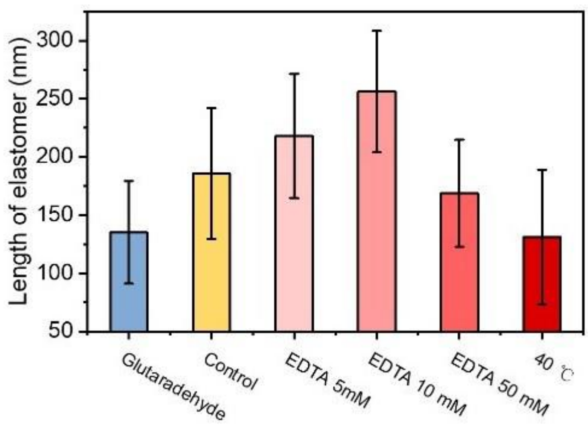

Fig. S8. Elastomer lengths after EPS treatments.

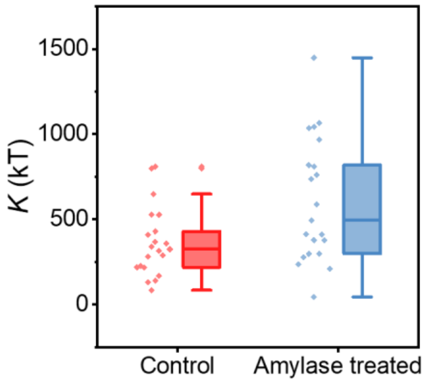

Fig. S9. Impact of amylase treatment on bacterial binding constant. After the amylase treatment, the distribution of bacterial binding constant became wider.

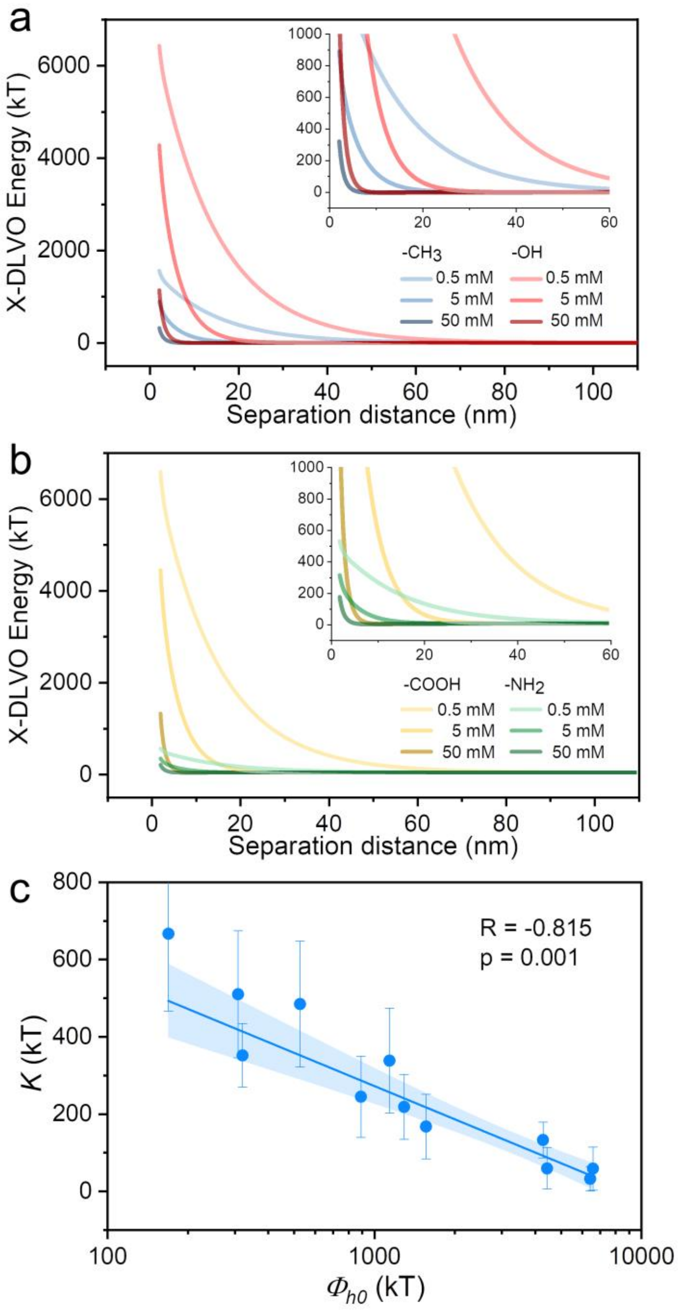

Fig. S10. X-DLVO energy profiles of bacteria under different adhesion conditions: C_{11}-CH_3 and C_{11}-OH (a), C_{11}-NH_2, and C_{11}-COOH (b). Binding constant as a function of X-DLVO energy at the position of the actual minimum separation distance (c).

Table. S1. Parameters used in X-DLVO calculation.

**Zeta potentials of different surfaces and *S. wittichii* RW1 (mV)**					
	$-CH_3$	-OH	$-NH_2$	-COOH	*S. wittichii* RW1
0.5 mM	-24.75	-22.09	-8.21	-30.71	-54.40
5 mM	-20.39	-23.71	-7.01	-33.69	-48.40
50 mM	-26.14	-28.46	-16.22	-37.36	-28.00
**Surface free energy components of model surfaces and *S. wittichii* RW1 (mJ/m²)**					
	$-CH_3$	-OH	$-NH_2$	-COOH	*S. wittichii* RW1
γ^{LW}	40.85	41.45	43.00	41.72	27.08
γ^{+}	0.28	0.47	0.28	0.18	0.59
γ^{-}	10.99	18.62	8.82	24.31	54.18

Table. S2. Calculated X-DLVO energy of bacteria-surface interaction

	0.5 mM		5 mM		50 mM	
	Φ_{h0} (kT)	Φ_{min1} (kT)	Φ_{h0} (kT)	Φ_{min1} (kT)	Φ_{h0} (kT)	Φ_{min1} (kT)
$-CH_3$	1558.89	-0.02	889.69	-0.24	320.17	-3.11
-OH	6427.81	-0.01	4275.31	-0.18	1137.93	-2.27
$-NH_2$	526.02	-0.02	308.32	-0.35	169.03	-4.06
-COOH	6581.37	-0.01	4428.86	-0.18	1291.48	-2.30

Reference

1. Gutman J, Herzberg M, & Walker SL (2014) Biofouling of reverse osmosis membranes: positively contributing factors of *Sphingomonas*. *Environ Sci Technol* 48:13941-13950.
2. Xing SF, *et al.* (2015) D‐Amino acids inhibit initial bacterial adhesion: thermodynamic evidence. *Biotechnol Bioeng* 112:696-704.
3. Mikelonis AM, Youn S, & Lawler DF (2016) DLVO approximation methods for predicting the attachment of silver nanoparticles to ceramic membranes. *Langmuir* 32:1723-1731.
4. van der Westen R, *et al.* (2018) Floating and tether-coupled adhesion of bacteria to hydrophobic and hydrophilic surfaces. *Langmuir* 34:4937-4944.
5. Porter MD, Bright TB, Allara DL, & Chidsey CE (1987) Spontaneously organized molecular assemblies. 4. Structural characterization of n-alkyl thiol monolayers on gold by optical ellipsometry, infrared spectroscopy, and electrochemistry. *J Am Chem Soc* 109:3559-3568.
6. Li S-W, Sheng G-P, Cheng Y-Y, & Yu H-Q (2016) Redox properties of extracellular polymeric substances (EPS) from electroactive bacteria. *Sci Rep* 6: 39098.
